# Do patients at high risk for Hepatitis C receive recommended testing? A retrospective cohort study of statewide Medicaid claims linked with OneFlorida clinical data

**DOI:** 10.1097/MD.0000000000028316

**Published:** 2021-12-17

**Authors:** Rahma S. Mkuu, Elizabeth A. Shenkman, Keith E. Muller, Tianyao Huo, Ramzi G. Salloum, Roniel Cabrera, Ali Zarrinpar, Emmanuel Thomas, Sarah M. Szurek, David R. Nelson

**Affiliations:** a Health Outcomes & Biomedical Informatics, College of Medicine, University of Florida, Gainesville, FL; b Department of Medicine, College of Medicine, University of Florida, Gainesville, FL; c Department of Surgery, College of Medicine, University of Florida, Gainesville, FL; d Department of Pathology, University of Miami Health System, Miami, FL.

**Keywords:** cancer screening, HCV, hepatitis C, hepatitis C testing, hepatocellular carcinoma

## Abstract

Hepatitis C virus (HCV) infection is a leading risk factor for hepatocellular carcinoma.

We employed a retrospective cohort study design and analyzed 2012–2018 Medicaid claims linked with electronic health records data from the OneFlorida Data Trust, a statewide data repository containing electronic health records data for 15.07 million Floridians from 11 health care systems. Only adult patients at high-risk for HCV (n = 30,113), defined by diagnosis of: HIV/AIDS (20%), substance use disorder (64%), or sexually transmitted infections (22%) were included. Logistic regression examined factors associated with meeting the recommended sequence of HCV testing.

Overall, 44.1% received an HCV test. The odds of receiving an initial test were significantly higher for pregnant females (odds ratio [OR]1.99; 95% confidence interval [CI] 1.86–2.12; *P* < .001) and increased with age (OR 1.01; 95% CI 1.00–1.01; *P* < .001).Among patients with low Charlson comorbidity index (CCI = 1), non-Hispanic (NH) black patients (OR 0.86; 95% CI 0.81–0.9; *P* < .001) had lower odds of getting an HCV test; however, NH black patients with CCI = 10 had higher odds (OR 1.41; 95% CI 1.21–1.66; *P* < .001) of receiving a test. Of those who tested negative during initial testing, 17% received a second recommended test after 6 to 24 months. Medicaid-Medicare dual eligible patients, those with high CCI (OR 1.14; 95% CI 1.11–1.17; *P* < .001), NH blacks (OR 1.93; 95% CI 1.61–2.32; *P* < .001), and Hispanics (OR 1.49; 95% CI 1.08–2.06; *P* = .02) were significantly more likely to have received a second HCV test, while pregnant females (OR 0.71; 95% CI 0.57–0.89; *P* = .003), had lower odds of receiving it. The majority of patients who tested positive during the initial test (97%) received subsequent testing.

We observed suboptimal adherence to the recommended HCV testing among high-risk patients underscoring the need for tailored interventions aimed at successfully navigating high-risk individuals through the HCV screening process. Future interventional studies targeting multilevel factors, including patients, clinicians and health systems are needed to increase HCV screening rates for high-risk populations.

## 1 Introduction

Hepatitis C virus (HCV) which spreads through contact with blood of an infected person, is the most common cause of chronic infectious disease death in the United States (US).^[1,2]^ Approximately 2.4 million people in the United States are living with the infection and its incidence has tripled since 2010.^[3]^ Chronic HCV infection affects the liver and is associated with the development of cirrhosis (ie, chronic liver damage), liver cancer, hepatic decomposition and death.^[4]^ Groups at increased risk of HCV infection include people who are current or former injection drug users, people diagnosed with a sexually transmitted infection or living with HIV, and recipients of infected blood and blood products before the advancement of blood screening methods.^[5]^ Currently, the primary mode of HCV infection in the United States is opioid-related drug use, which is specifically associated with raising HCV infection rates among younger persons.^[6,7]^ The annual rate of acute HCV infection increased threefold between 2018 and 2019 and was highest among persons aged 20 to 39 years’ old.^[6]^

A growing body of literature demonstrates that people covered by public insurance, including Medicaid-insured individuals, have higher rates of HCV infection because of a confluence of interconnected behavioral (eg, substance use disorder), economic (eg, poverty), and health risk factors (eg, sexually transmitted infections, co-occurring health conditions).^[8,9]^ Although HCV screening is associated with early detection and increased survival from HCV-related complications,^[10]^ little attention has been given to understanding HCV testing guideline concordance among individuals who are receiving Medicaid and are at highest risk for HCV infection.

The Florida Medicaid population is an ideal cohort to study individuals at high-risk for HCV infection. Florida is the third largest state and has the fourth largest Medicaid program in the United States, covering 1 in 9 adults between the ages of 19 to 64. The state is also among the most ethnically and geographically diverse.^[11,12]^ Florida is ranked third nationally with the highest number of persons living with HCV.^[13]^ Furthermore, Florida has among the highest rates of individuals with risk factors for HCV infection including HIV, opioid use, and syphilis.^[14–16]^ The synergistic interactions of interrelated HCV risk factors among the Medicaid population is reflected in a recent nationally representative study.^[9]^ According to the study, women covered by public insurance (Medicaid or Medicare) are 5.5 times more likely to be diagnosed with HCV, and women with opioid use disorder are 6.4 times more likely to be diagnosed with HCV. However, women with both public insurance and opioid use disorder experience compounding vulnerability for HCV infection resulting in a 9.9 times higher risk of being infected.^[9]^

Screening rates for HCV are suboptimal overall but particularly among populations at high-risk for HCV infection.^[17–19]^ High-risk individuals should be screened periodically, with annual testing recommended for all persons who inject drugs and for men infected with HIV who have unprotected sex with men.^[20,21]^ For other high-risk individuals, there is limited information about the optimal repeat testing frequency, leaving the periodicity to the clinician's discretion. The sequence of recommended testing for those who are at high-risk entails: initial HCV-antibody testing with reflex HCV RNA polymerase chain reaction (PCR) testing for those with a positive antibody result; and for those with a negative initial HCV antibody testing result, follow-up HCV antibody and/or HCV RNA testing after 6 months should be performed.^[21]^ Little is known about whether populations at high-risk for HCV infection receive recommended initial and repeat screening. For such populations, a focus on adhering to screening guidelines is critical for achieving improved health outcomes and quality of care. For example, Medicaid patients experience a higher number of HCV-related emergency department visits likely because of severe complications associated with delayed HCV diagnosis.^[22]^

The purpose of this retrospective observational study is to quantify the relationship between receipt of HCV screening and health and sociodemographic factors, for individuals with Medicaid coverage who are at high-risk for having HCV infection. The analysis specifically includes interrogating the receipt of antibody, RNA and repeat HCV testing. This study leverages Medicaid claims data linked to electronic health records and laboratory data from the OneFlorida Data Trust. The Data Trust contains the health records for 15.07 million Floridians from health care systems throughout the state.^[23]^

This study has 2 aims:

1. To characterize the individuals in Medicaid at higher risk for HCV infection due to HIV/AIDs, substance use disorder, or sexually transmitted infections (STIs) in terms of age, sex, race, ethnicity, place of residence (urban, rural), social vulnerability, and health status. Hypothesis: Individuals with high social vulnerability including racial/ethnic minorities, rural populations and those with multiple chronic conditions will make up a larger percentage of individuals who are high-risk for HCV infection.
2. To quantify among these high-risk individuals, the associations between health status (presence of co-occurring conditions and/or pregnancy) and sociodemographic characteristics (age, sex, race, ethnicity and social vulnerability) on the one hand, and the receipt of initial HCV antibody testing, receipt of an HCV RNA confirmation test if positive during initial HCV antibody testing, and receipt of a second HCV-antibody test 6 months after an initial negative HCV antibody test. Hypothesis: Overall, a suboptimal HCV screening rate will be observed in the high-risk group. Individuals with high social vulnerability will have lower HCV screening rates.

## 2 Materials and methods

### 2.1 Data sources

We analyzed secondary data from the OneFlorida Data Trust, which is a secure and centralized data repository containing collated electronic health records data for 15.07 million Floridians from 11 health care systems across the state and the Florida Medicaid Program.^[23]^ OneFlorida Data are available through requests made to the OneFlorida Coordinating Center. All requests undergo scientific review and also must have IRB approval before data are provided. The OneFlorida Data Trust is the informatics infrastructure that supports pragmatic trials, comparative effectiveness research, implementation science, and other research in the OneFlorida Clinical Research Consortium.^[23]^

A data use agreement between the University of Florida and the Florida Medicaid program allows the OneFlorida Data Trust to link Medicaid enrollment and claims data with the electronic health record information. The data are HIPAA-limited data sets, which restrict the types of protected health information to include only dates (eg, birthdates and dates of service) and location (5 or 9 digit zip code level, which allows for geocoding). Data are submitted to the Data Trust by the health care system partners and harmonized using the Patient Centered Outcomes Research Institute's Common Data Model.^[24]^

### 2.2 Participants

We derived the study population from Medicaid linked with EHR data in the OneFlorida Data Trust between 2012 and 2018. The analytic sample for this study included all individuals who were 18 years of age and older and met the criteria of being at high-risk for HCV infection by having either diagnosis of HIV/AIDS, diagnosis of a substance use disorder, or diagnosis of an STI including chlamydia, gonorrhea, pelvic inflammatory disease, syphilis, and trichomoniasis. We identified high-risk diagnosis by using *International Classification of Diseases ICD-9, Ninth Revision, Clinical Modification* or ICD-10-CM diagnosis codes. Individuals were required to have two outpatient visits in a 12-month period or 1 inpatient stay with the relevant *ICD* codes. We included in our analysis the records of 30,113 patients who met these criteria.

### 2.3 Outcomes

Given the importance of follow-up confirmatory screening for individuals at high-risk for HCV infection,^[21]^ we assessed the following outcomes related to concordance with screening guidelines: received initial HCV antibody test, received RNA testing if initial HCV antibody test was positive and received follow-up HCV antibody test 6 to 24 months after initial HCV antibody test if it was negative. Patients with missing data regarding HCV screening were not included in this study as to limit bias assumptions of differentiating whether a person truly did not get screened or the a person got screening but the data were not in the system.

Receipt of the initial HCV antibody test was determined to have occurred if patients met any of the following criteria: had an HCV antibody lab result or a procedure code (40.6%), or had an HCV RNA lab procedure code or lab result (2.1%), or had an HCV diagnosis, or received a direct acting antiviral (DAA) to treat HCV without evidence of any HCV antibody and/or HCV RNA testing (1.4%). A diagnosis of HCV was determined based on the documentation of the diagnosis in ≥2 outpatient visits or one inpatient admission within 18 months. Diagnoses associated with ancillary services such as radiology or laboratory tests were excluded. The receipt of a DAA was determined using pharmacy claims information. Only 415 (1.4%) individuals were assumed to have received previous HCV antibody and HCV RNA testing based on documentation of an HCV diagnosis or receipt of DAAs.

Receipt of RNA testing was determined to have occurred if the initial HCV antibody test was positive and if patients met any of the following criteria: had an RNA lab result, or had an RNA test procedure code, or had an HCV diagnosis or DAA treatment as previously described. A total of 2749 patients met the criteria for receipt of RNA testing after a positive initial antibody test.

Receipt of a second HCV antibody test after an initial negative HCV antibody test was determined if the patient had an initial negative HCV antibody test followed by a second HCV antibody test within 6 to 24 months post the initial test. A total of 4350 patients who had a negative result for the initial HCV antibody test were included.

### 2.4 Predictor variables

Predictor variables included age, race/ethnicity, sex (male, female), pregnancy status in females (pregnant, not pregnant), Charlson comorbidity index (CCI), presence and number of behavioral health (BH) conditions (ie, substance use and mental health), number of months enrolled in Medicaid, Medicaid-Medicare dual eligibility, and the social vulnerability index (SVI).

The CCI weights the relevance of 17 comorbidities in predicting 1-year mortality using International Classification of Diseases (*ICD-10*) codes and is widely utilized in cancer health services research as a reflection of health status.^[25]^ To calculate the CCI, we calculated comorbidities based on the 1 year preceding the presentation of the high-risk diagnosis that qualified the individual for study inclusion. The Centers for Disease Control and Prevention (CDC) SVI ranks census tracts on 15 social factors, including poverty, lack of vehicle access, and crowded housing. We used 9-digit zip code information to categorize each individual's social vulnerability, which is reported on a scale of 0 to 1 with higher scores indicating greater vulnerability.^[26]^

### 2.5 Ethical statement

The University of Florida Institutional Review Board approved the protocol of this study (#IRB201901953). The work was supported in part by the OneFlorida Clinical Data Network, funded by Patient-Centered Outcomes Research Institute PCORI CDRN-1501-26692.

## 3 Statistical analysis

### 3.1 Split sample approach

We first conducted exploratory analyses using a split-sample approach^[27]^ whereby a test sample set was used as a means of avoiding bias in prediction accuracy and checking for data sensitivity. The whole sample (n = 30,113) was split into 2 parts, an exploratory sample (*n* = 7618) and a confirmatory sample (*n* = 22,495), stratified by race/ethnicity (NH white, NH black, Hispanics, other). The split sample included 25% NH white, NH black and Hispanics, and 50% of those with other races/ethnicities. Exploratory analyses allowed for refining outcome definitions, refining algorithms used to compute the outcomes and predictors, and developing and refining the logistic regression models. After we finalized the models, we tested the models on the confirmatory sample. Finally, we combined the exploratory and confirmatory samples to estimate the model coefficients on the combined total sample.

### 3.2 Analysis

All the statistical analyses were conducted using SAS 9.4 (SAS Institute, Cary, NC). Differences were considered statistically significant if *P* value ≤.05. We used descriptive statistics to characterize the population sample by race and ethnicity. We used logistic regression models to evaluate the association between the predictors and outcomes since all the outcomes of interest are binary. All predictor variables from the exploratory analysis were included in the full-sample models. We also accounted for interaction effects by including two-way and three-way interactions between race/ethnicity, male sex (M), non-pregnant female (F), and pregnant female (P) (MFP), and CCI, as well as 2- and 3-way interactions between race/ethnicity, MFP, and BH. The full model is shown below:


$$\begin{array}{l} \text{Outcome}=\text{Age Race}/\text{ethnicity MFP Race}/\text{ethnicity}\times \text{MFP}\\ \;\text{CCI CCI}\times \text{Race}/\text{ethnicity CCI}\times \text{MFP CCI}\times \text{Race}/\text{ethnicity}\times \text{MFP}\\ \;\text{BH BH}\times \text{Race}/\text{ethnicity BH}\times \text{MFP BH}\times \text{Race}/\text{ethnicity}\times \text{MFP}\\ \;\text{Months Enrolled Dual Eligible SVI} \end{array}$$

During exploratory analysis, we used the split sample to check collinearity among the predictors and dropped terms that had high collinearity (conditional index >11). We then conducted backward groupwise model selections to drop predictors and interaction terms that were non-significant for each outcome variable. After finalizing the regression models, we tested the model performance on the confirmatory sample. Finally, we re-fit the model on the pooled final sample and report the *P* values, odds ratios (OR) and confidence intervals (CIs) estimated from the final sample. The results, including AUC, *P* values and ORs from the regression analysis were similar for the exploratory and the final analysis, suggesting the robustness of the models.

## 4 Results

### 4.1 Population characteristics

Among the 30,113 adults included in the final sample, the majority of the high-risk sample (64.6%) had a substance use disorder, 22% had a STI and 20% were diagnosed with HIV. Demographic characteristics of the sample are provided in Table 1. Overall, 44.1% of the sample received an initial HCV antibody test of which 21.2% were positive. Of those who tested positive after initial HCV antibody testing, 96.7% received RNA testing. Among those who tested negative during the initial HCV antibody test, 17.1% received a second HCV antibody test 6 to 24 months after receiving the initial test.

**Table 1:** Sample characteristics, Florida Medicaid adults with linked OneFlorida clinical data, 2012–2018 (N = 30,113).

		Race/Ethnicity			
	Total N = 30,113	Hispanic n = 3540	Non-Hispanic Black/AA n = 12,635	Non-Hispanic White n = 13,585	Other n = 353
	N (%)	n (%)	n (%)	n (%)	n (%)
Age	36.0 ± 9.2	35.4 ± 9.7	35.5 ± 9.4	36.7 ± 8.8	36.0 ± 9.4
Sex and pregnancy					
Pregnant female	26.3%	21.7%	27.1%	26.9%	20.4%
Nonpregnant female	38.4%	37.0%	38.6%	38.5%	37.7%
Male	35.3%	41.2%	34.2%	34.6%	41.9%
HCV risk factors					
HIV	20.0%	20.1%	32.5%	8.3%	23.2%
STI	22.0%	27.8%	32.1%	11.3%	17.8%
Substance use disorder	64.6%	58.5%	44.9%	84.6%	63.7%
Social Vulnerability Index quartiles					
4 (Most vulnerable)	29.0%	31.3%	43.2%	15.5%	22.9%
3	23.9%	23.0%	22.2%	25.6%	26.6%
2	18.8%	19.5%	11.2%	25.6%	21.5%
1 (Least vulnerable)	11.4%	9.9%	5.5%	17.1%	17.0%
Undetermined	16.9%	16.3%	17.9%	16.2%	11.9%
Dual eligible	15.3%	13.3%	16.8%	14.3%	18.4%
Charlson Comorbidity Index^∗^	2.2 ± 3.0	2.1 ± 3.0	2.9 ± 3.4	1.5 ± 2.5	2.3 ± 2.9
Average months enrolled in Medicaid	52.5 ± 24.4	52.1 ± 4.3	56.7 ± 23.6	48.7 ± 24.5	50.9 ± 24.4
Had initial HCV Antibody test only	44.1%	43.1%	46.9%	41.7%	44.5%
Had follow-up RNA Test after Initial Posive HCV test^†^	96.7% (n = 2749)	96.3% (n = 246)	96.3% (n = 380)	96.9% (n = 2099)	91.7% (n = 24)
Had at least 1 HCV antibody test 6–24 mo Following an Initial Negative Test^‡^	17.1% (n = 4350)	16.8% (n = 340)	20.6% (n = 2380)	11.8% (n = 1572)	17.2% (n = 58)

HCV = hepatitis C virus, STI = sexually transmitted infection

∗ The Charlson Comorbidity Index was calculated as a weighted Sum of 17 Charlson Comorbidity Groups.

† The denominator is the number of participants who had a positive initial HCV antibody test in each category.

‡ The denominator is the number of participants who had a negative initial HCV antibody test in each category.

### 4.2 Initial HCV antibody test

**Figure 1:**
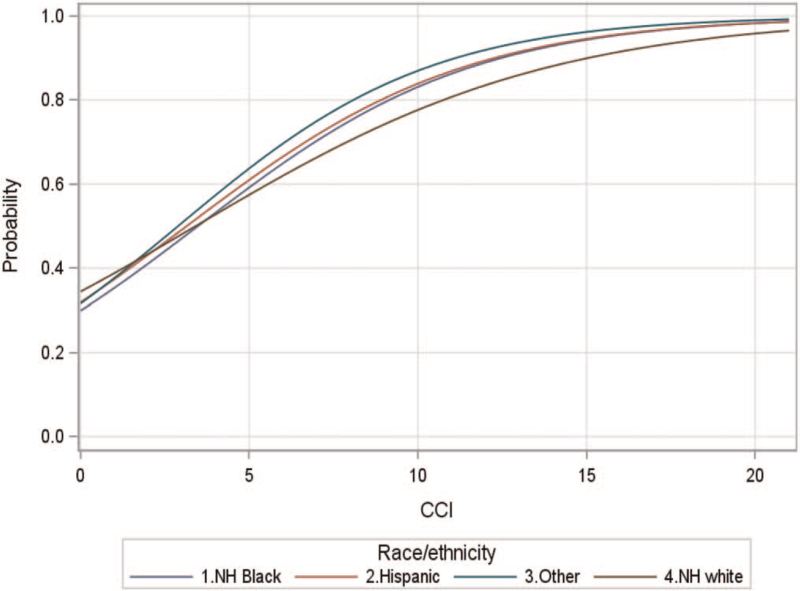
The predicted probably of receiving HCV antibody test among Florida Medicaid members by race/ethnicity and CCI. There was a significant interaction between race/ethnicity and CCI illustrated by the interaction curves.

Significant predictors of receiving an initial HCV antibody test in the high-risk population included age, sex, race/ethnicity, and the CCI. The regression models had an overall AUC of 0.69, indicating moderate model fit. For every 1 year increase in age, there was 0.6% increased odds of receiving initial HCV antibody testing. Pregnant women had almost two times higher odds (OR 1.99; 95% CI 1.86–2.12; *P* < .001) of receiving the initial HCV antibody test compared to non-pregnant females. Males had significantly lower odds of receiving the initial HCV antibody tests compared to nonpregnant females (OR 0.881; 95% CI 0.835–0.93; *P* < .001). There was a significant interaction between race/ethnicity and CCI illustrated by the interaction curves showing the predicted probably of receiving HCV antibody test among Florida Medicaid members (Fig. 1).

Among individuals with CCI = 1 (median CCI), NH black patients had 14% lower odds of receiving initial HCV antibody testing compared to NH white patients. However, among those with higher CCI (CCI = 10), NH black patients had 42% higher odds of receiving the initial HCV antibody test compared to NH white. The odds of receiving the initial HCV antibody test were also higher among Hispanic compared to NH white patients when CCI = 10 (OR 1.501; 95% CI 1.174–1.918; *P* = .0012) (Table 2).

**Table 2:** Odds of receiving initial HCV antibody test.

Effect	Odds ratio	95% Confidence interval		*P*
Age	1.006	1.004	1.009	<.001
Sex				
Nonpregnant female	1	0	0	
Males	0.881	0.835	0.93	<.001
Pregnant female	1.986	1.864	2.115	<.001
Race/ethnicity, CCI = 1				
NH white	1	0	0	
NH black	0.86	0.813	0.911	<.001
Hispanic	0.941	0.865	1.023	.154
Other	0.952	0.743	1.22	.698
Race/ethnicity, CCI = 4				
NH white	1	0	0	
NH black	1.016	0.952	1.085	.628
Hispanic	1.099	0.995	1.214	.062
Other	1.203	0.916	1.58	0.183
Race/ethnicity, CCI = 10				
NH white	1	0	0	
NH black	1.418	1.209	1.663	<.001
Hispanic	1.501	1.174	1.918	.001
Other	1.922	0.964	3.832	.064

CCI = Charlson comorbidity index calculated as a weighted sum of 17 Charlson Comorbidity Groups.

### 4.3 Second HCV antibody test after initial negative HCV antibody test

The AUC for the final model examining the probability of having a second HCV antibody test 6 to 24 months after the initial negative HCV antibody test was 0.66, indicating moderate model fit. Non-Hispanic blacks (OR 1.93, 95% CI 1.61–2.32; *P* < .0001) and Hispanics (OR 1.49; 95% CI 1.08–2.06; *P* = .015) had higher odds of getting the second HCV antibody test compared to NH whites. Those who were Medicaid-Medicare dual-eligible had 39% higher odds of having the second test (OR 1.39; 95% CI 1.143-1.70; *p* = 0.001). Having comorbid behavioral health conditions also increased the odds of receiving the second HCV antibody test. Each additional behavioral health condition resulted in a 13% increased odds of getting the second HCV antibody Test. Increased CCI score was associated with higher odds of receiving the second test (OR 1.14; 95% CI 1.11–1.17; *P* < .001). Pregnant females had 29% lower odds of receiving the second HCV antibody test compared to non-pregnant females (OR 0.71; 95% CI 0.57–0.89; *P* = .002). The odds of receiving the second HCV antibody test also increased with the number of months that patients were enrolled in Medicaid (OR 1.01; 95% CI 1.00–1.01; *P* = .009). There was no significant difference in the probability of getting the second test between males and nonpregnant females (Table 3).

**Table 3:** Odds of receiving second HCV antibody test 6 to 24 months after negative initial HCV antibody test.

Effect	Odds ratio	95% Confidence interval		*P*
Age	0.993	0.985	1.002	.128
Race/ethnicity				
NH white	1	0	0	
NH black	1.929	1.607	2.315	<.001
Hispanic	1.494	1.081	2.064	.015
Other	1.554	0.773	3.125	.216
Sex				
Nonpregnant female	1	0	0	
Male	0.883	0.738	1.057	.177
Pregnant female	0.708	0.565	0.886	.003
Charlson Comorbidity Index	1.138	1.11	1.168	<.001
Behavioral health conditions	1.128	1.067	1.192	<.001
Average months enrolled in Medicaid	1.005	1.001	1.008	.009
Dual eligible	1.394	1.143	1.698	.001

### 4.4 HCV RNA test after initial positive HCV antibody test

Among those who tested positive during the initial HCV antibody test, 96.7% received RNA testing. The high compliance rate of screening limited our ability to analyze a model examining disparity.

## 5 Discussion

To our knowledge, our study is the first to employ a longitudinal retrospective study design to characterize a Medicaid population at high-risk for HCV infection and to examine the receipt of recommended HCV testing and follow-up using Medicaid claims linked with electronic health record data. Overall, we observed that more than half of the high-risk population did not receive initial HCV antibody testing consistent with studies reporting suboptimal testing in high-risk populations.^[28]^ We observed a higher rate of receiving HCV RNA confirmatory testing (96.73%) after a positive initial HCV antibody test compared to other studies,^[29,30]^ with some studies reporting rates as low as 20%.^[31,32]^ The key clinically relevant finding was that only 17% received a second antibody test 6 to 24 months following negative results from initial HCV antibody testing.

Whereas HCV screening has been consistently recommended for high-risk groups, the US Preventive Health Task Force only recently revised their guidelines (in 2020) to expand its recommendation for 1-time universal screening to cover all adults aged 18 to 79 years.^[33]^ Despite previous emphasis to screen high-risk populations, high-risk patients received suboptimal HCV screening during the 2012 to 2018 time period observed in this study. The initial HCV antibody and follow-up RNA testing rates in the observed high-risk Florida Medicaid population is higher than the rates observed in previous studies of patients regardless of insurance status.^[19]^ These higher rates may be attributed to the high prevalence of HCV in Florida which may lead to more awareness of screening and recommendations among both patients and providers.^[13]^ Further, Medicaid-insured patients have higher screening rates compared to their privately insured counterparts; therefore the higher screening rates observed may be associated with Medicaid enrollment.^[34]^

We found that pregnant women had higher odds of receiving initial HCV antibody testing but lower rates of receiving a second test after 6 to 24 months of initial testing. The findings raise intriguing questions regarding follow-up care for high-risk pregnant women covered by Florida Medicaid, which finances 46.7% of births in the state.^[35]^ Growing evidence shows increasing HCV prevalence among pregnant women and women of reproductive age, resulting in recommendations for universal HCV screening for pregnant women.^[18,20,36]^ HCV is associated with deleterious maternal and infant health outcomes, most notably vertical transmission of HCV, which is the leading cause of HCV infection among children.^[37]^ Therefore, screening pregnant women for HCV is imperative to identify at-risk infants who may need follow-up monitoring.^[8,37]^ We found that pregnant women at high-risk for HCV who received an initial negative HCV antibody test had significantly lower odds of receiving a second HCV antibody test 6 to 24 months after the initial test. The decreased likelihood of receiving the second test is likely associated with loss of insurance coverage. During the observation period for this study, Medicaid coverage for pregnant women ceased 60 days post-delivery and the disruption in health coverage likely explains, at least partially, the lack of follow-up screening for the second HCV antibody test.^[38]^ In our study, the odds of receiving the second HCV antibody test increased with the number of months a patient was enrolled in Medicaid.

Among individuals at high-risk for HCV who were not pregnant and did not have co-occurring conditions, NH black patients had 14 times lower odds of receiving the initial HCV antibody test compared to NH white patients, which is consistent with the literature reporting lower screening rates among NH black compared to NH white patients.^[17]^ Contrary to expectations, we observed that among high-risk adults with co-occurring conditions, NH black and Hispanic individuals had higher odds of receiving an initial HCV antibody test. NH black and Hispanic patients also had higher odds of receiving a second antibody test 6 to 24 months after initial negative test results compared to NH whites. These results may be explained in part by increased efforts to reduce longstanding disparities in screenings for racial and ethnic minorities, particularly for those who come into frequent contact with the healthcare system due to the presence of co-occurring conditions.^[39]^

Overall, our results highlight a significant need to improve HCV screening navigation for high-risk individuals in Medicaid who have an initial negative HCV antibody screening result. Care navigation intervention programs including Project INSPIRE, Check Hep C and End Hep C SF train peer and patient navigators to provide HCV outreach, prevention, linkage to care, and coordination services including screening, treatment, and reinfection prevention after a patient is cured.^[29,40]^ For high-risk individuals, programs like End Hep C SF provide HCV services in nonclinical settings including residential drug treatment programs, mobile outreach, sexual health clinics, and syringe access programs.^[41]^ Navigation programs such as these have the potential to improve follow-up screening rates for high-risk populations and the opportunity to treat HCV, which is critical for the prevention of further disease including HCV.^[40]^

Despite significant advancements in the efficacy and availability of HCV treatment, the high cost of HCV treatment continues to be a major barrier to access to care.^[42]^ Policies that restrict access to HCV treatment, such as requiring abstinence from alcohol and other substances, may limit physicians from recommending screening if treatment will be restricted.^[43]^ For example, given the high cost of treatment, Medicaid programs have traditionally limited access to HCV treatment by using prior authorization policies.^[44]^ As a result of prior authorization, more than half of patients referred to treatment are not prescribed antivirals because they do not meet treatment eligibility criteria due to uncontrolled co-occurring conditions, inability to follow treatment recommendations, substance use disorder, fibrosis stage, or treatment refusal.^[45]^

At the policy-level, 25 states presently require abstinence from substance use ranging from 1 to 12 months as an eligibility criterion.^[46]^ Some private insurers have similar restrictions. Florida Medicaid requires at least 1 month of sobriety from alcohol and substance use with a confirmed negative urine test or blood rest before initiation of treatment.^[47]^ Given that the majority of the high-risk population in the present study had a substance use disorder, sobriety restrictions may explain the low rates of receiving recommended initial and follow-up testing. Patients who are not adherent to sobriety requirements may be discouraged from seeking follow-up testing. Interventions are needed to address patient-level modifiable barriers to treatment such as substance use disorder that may preclude patients from seeking HCV testing. Florida Medicaid also requires that antiviral prescriptions are provided in consultation with specialized clinicians (eg, hematologist, gastroenterologist, infectious disease specialist or transplant specialist), which may be challenging for residents of medically underserved areas.^[48]^ Providers who are not specialized report low competency related to HCV management and limited expertise in treating high-risk patients, resulting in misperceptions and stigma towards patients.^[49]^ Improving provider knowledge of HCV, and expanding options for nonspecialist providers to prescribe antivirals have the potential to improve screening rates.^[49]^

This study has some limitations. Our focus was on the Medicaid population and the results may not be generalizable to individuals with commercial insurance, Medicare, or the uninsured. Additionally, we had limited access to patient information to capture risk behaviors, particularly related to present substance use disorder. We relied on diagnostic codes to identify substance use disorder, which likely resulted in an underestimation of individuals with this condition.

Notwithstanding these limitations, strengths of our study include utilization of Medicaid claims linked with electronic health record data from the OneFlorida Data Trust which contains a robust, diverse, and sizable population of interest, allowing for precise characterization. The database has been used in previous studies to provide a statewide landscape addressing several distinct medical conditions.^[50]^

We recommend that future studies examine the influence of provider specialty (eg, primary vs specialty care) on screening rates and subsequent referral to specialists for positive test results. Examining both provider- and patient-level factors that influence HCV screening and follow-up will allow for the development of tailored multi-level interventions.

In conclusion, our analysis using a large population database of high-risk Medicaid insured individuals demonstrates suboptimal HCV screening and substantial attrition in recommended follow-up screening. Understanding the factors associated with not meeting HCV screening recommendations has clinical significance for patients, clinicians, and researchers. Researchers can develop interventions to address factors associated with under screening for HCV for high-risk populations that should receive screening, and future patients will benefit from developed interventions that aim to improve screening rates. Increasing HCV screening has the potential to improve HCV-related patient outcomes through increasing treatment rates among those who are infected with HCV and subsequently limiting its transmission. Examining HCV screening in the Florida Medicaid population has implications to inform Medicaid policy and quality of care initiatives targeting Medicaid beneficiaries who are at high-risk of having HCV infection in similar settings. The findings have important implications for both the clinical and scientific community as they emphasize the need to further identify provider- and patient-level factors that influence HCV screening and follow-up, and the need to develop multilevel interventions to support HCV screening and improve care navigation among highrisk populations.

## Author contributions

**Conceptualization:** Elizabeth A Shenkman, Keith E Muller, Tianyao Huo, Ramzi G Salloum, Roniel Cabrera, Ali Zarrinpar, Emmanuel Thomas, Sarah M Szurek, David R Nelson.

**Data curation:** Tianyao Huo.

**Formal analysis:** Tianyao Huo.

**Funding acquisition:** Elizabeth A Shenkman.

**Investigation:** Elizabeth A Shenkman, Tianyao Huo, David R Nelson.

**Methodology:** Keith E Muller, Tianyao Huo.

**Project administration:** Rahma S Mkuu.

**Resources:** Elizabeth A Shenkman.

**Supervision:** Elizabeth A Shenkman, Keith E Muller.

**Validation:** Keith E Muller.

**Visualization:** Tianyao Huo.

**Writing – original draft:** Rahma S Mkuu.

**Writing – review & editing:** Elizabeth A Shenkman, Keith E Muller, Ramzi G Salloum, Roniel Cabrera, Ali Zarrinpar, Emmanuel Thomas, Sarah M Szurek, David R Nelson.
